# Exocytosis and Endocytosis in Neuroendocrine Cells: Inseparable Membranes!

**DOI:** 10.3389/fendo.2013.00135

**Published:** 2013-10-02

**Authors:** Sébastien Houy, Pauline Croisé, Olga Gubar, Sylvette Chasserot-Golaz, Petra Tryoen-Tóth, Yannick Bailly, Stéphane Ory, Marie-France Bader, Stéphane Gasman

**Affiliations:** ^1^Institut des Neurosciences Cellulaires et Intégratives (INCI), Centre National de la Recherche Scientifique (CNRS UPR 3212), Université de Strasbourg, Strasbourg, France

**Keywords:** exocytosis, compensatory endocytosis, membrane lipids, neuroendocrine cells, chromaffin cells

## Abstract

Although much has been learned concerning the mechanisms of secretory vesicle formation and fusion at donor and acceptor membrane compartments, relatively little attention has been paid toward understanding how cells maintain a homeostatic membrane balance through vesicular trafficking. In neurons and neuroendocrine cells, release of neurotransmitters, neuropeptides, and hormones occurs through calcium-regulated exocytosis at the plasma membrane. To allow recycling of secretory vesicle components and to preserve organelles integrity, cells must initiate and regulate compensatory membrane uptake. This review relates the fate of secretory granule membranes after full fusion exocytosis in neuroendocrine cells. In particular, we focus on the potential role of lipids in preserving and sorting secretory granule membranes after exocytosis and we discuss the potential mechanisms of membrane retrieval.

## Introduction

Mammalian cells exhibit complex and dynamic patterns of intracellular membrane traffic between various organelles. Although much has been learned concerning the mechanisms of vesicle transport and vesicle fusion at donor and acceptor compartments, relatively little attention has been paid to understanding how organelle homeostasis is maintained. This aspect is particularly important in neurosecretory cells in which intense membrane trafficking and mixing occurs between secretory vesicles and the plasma membrane during secretion of various transmitters, peptides, and hormones. Calcium-regulated exocytosis, i.e., fusion of secretory vesicles with the plasma membrane results in the merging of these two membrane compartments, hence triggering an increase in plasma membrane surface and loss of identity. As a consequence, exocytosis must be coupled to a compensatory endocytotic process allowing the plasma membrane to recover its integrity and the granule membrane to be recycled. In neurons, molecular mechanisms of synaptic vesicle recycling and coupling with exocytosis have been intensively studied, but is still debated [for reviews see (1–3)]. However, the equivalent process for large dense core granules in neuroendocrine cells remains largely unexplored.

**Figure 1:**
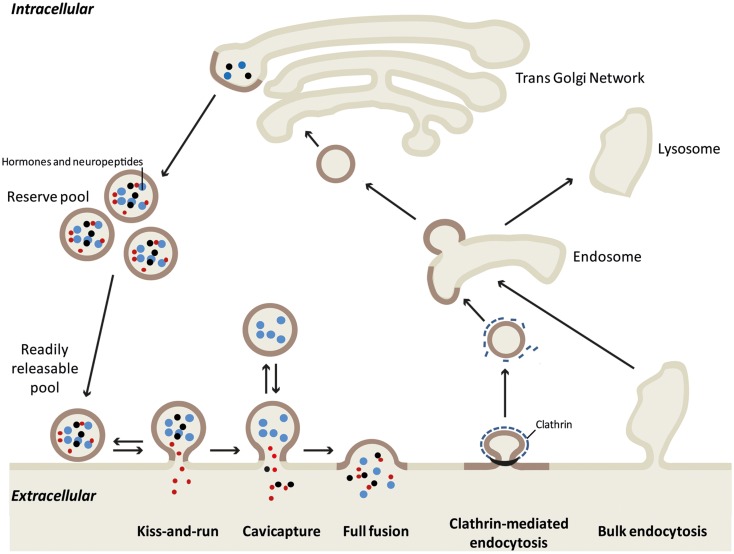
**Different models of exo-endocytosis coupling in neuroendocrine cells**. Hormone sorting and large dense core granule biogenesis occurs at the trans-Golgi network. Mature granules either constitute the reserve pool or are recruited to the plasma membrane as a readily releasable pool. Large proteins (blue dots), small neuropeptides (black dots), and small molecules like catecholamines (red dots) can be released differentially according to the exo-endocytosis mode. During “kiss-and-run” mode, only small molecules are released through a narrow fusion pore, whereas cavicapture (granule cavity capture) allow the partial release of small neuropeptides (7). Note that for these two modes, retrieval of intact granules is easily conceived as the granule shape remains almost intact. During full fusion exocytosis, the intra-granular contents are all released and the granule membrane collapses into the plasma membrane. This membrane incorporation is compensated by clathrin-mediated endocytosis that specifically retrieves granule membrane piece by piece [see Figure 2 and (19)]. After uncoating, the endocytic granule membrane reaches early endosome where granule components remain clustered (12), before being re-maturated at the Golgi network. During intense exocytotic activity, bulk endocytosis supports clathrin-mediated endocytosis by internalizing large plasma membrane invaginations that most likely follow the lysosomal degradation pathway.

In neuroendocrine cells, secretion can occur through different modes of exocytosis depending on the physiological demand (see Figure 1 for details). The “kiss-and-run” mode allows only the release of catecholamines and other small molecules through a narrow fusion pore (4, 5). During “cavicapture” (granule cavity capture) mode, expansion of the fusion pore triggers partial release of the small proteins (6–9). During kiss-and-run and cavicapture processes, the granule shape remains almost intact, whereas during “full collapse” or “full fusion” exocytosis, granules lose their round shape, flatten out in the plane of the plasma membrane leading to the merging of these two compartments and the complete release of the granular content (10–12). Whereas the molecular mechanisms of the various exocytotic modes (secretory vesicle recruitment, docking, priming, and fusion processes) have been largely explored, how granule and plasma membranes maintain their composition and recover their integrity after full fusion exocytosis is poorly known. Two types of retrieval have been described after full fusion exocytosis: clathrin-mediated endocytosis and bulk endocytosis. Bulk endocytosis occurs during elevated secretory activity when clathrin-mediated endocytosis is unable to fully compensate the large increase in membrane surface. To rapidly reverse this excess of plasma membrane, bulk endocytosis internalizes large invaginations of plasma membrane, which then form endosomal-like compartments. Bulk endocytosis has been described in several reviews (13, 14). Here, we focus on potential mechanisms that allow neuroendocrine cells to compensate full fusion exocytosis of large dense core vesicles through clathrin-mediated endocytosis.

## The Fate of the Secretory Granule Membrane after Fusion

For a long time, it was believed that after full fusion exocytosis vesicular components diffuse into the plasma membrane and are subsequently randomly internalized. This model implies that both the secretory vesicle and plasma membranes lose their identities and that exocytosis is not directly coupled to endocytosis. In contradiction with this model, synaptic activity in neurons results neither in the overall dispersion of vesicle components in the plasma membrane nor in the enrichment of plasma membrane components in synaptic vesicles (2). Similarly, despite full fusion exocytosis in neuroendocrine cells, granules, and plasma membranes seem to maintain their specific protein composition.

Early evidence for exo-endocytosis coupling came from morphological studies in the 80s suggesting that large dense core granule membrane-bound components could be retrieved after exocytosis (10, 15). At the same time, Geisow and co-workers observed an important increase in the number of coated pits containing secretory granule components in secretagogue-stimulated chromaffin cells (16). Later on patch-clamp and imaging studies suggested a fast temporal coupling between exocytosis and endocytosis processes (17, 18).

Using electron microscopy of cultured chromaffin cells, our group has recently described clustering of secretory granule proteins on the plasma membrane after full fusion exocytosis, arguing against the idea that granule components are dispersed in the plasma membrane (12). The group of Holz recently confirmed that chromaffin granule markers remain associated after fusion (19). Additionally, we have shown that these granule-bound proteins are subsequently internalized through vesicles devoid of plasma membrane makers (12). In other words, granule membranes are maintained together as “microdomains” after exocytosis and are subsequently recaptured without intermixing with the plasma membrane. How do neuroendocrine cells preserve granule membrane integrity after full collapse and precisely sort granule membrane lipids and associated proteins?

## Lipids as Central Organizers of Exo-Endocytosis Coupling?

### Creating membrane domains to preserve granule identity

The preservation of secretory granule identity after fusion with the plasma membrane implies that both proteins and lipids do not diffuse in the plasma membrane. Lateral segregation of membrane lipids would in this case represent an obvious sorting mechanism. Interestingly, we and others have shown that exocytosis requires several types of lipid remodeling processes (described in the present “Research Topic” by Amar et al. (28)). Some of these processes might contribute to prevent granular component from diffusing. For instance, secretagogue-evoked stimulation of chromaffin cells triggers the formation of lipid raft microdomains at the plasma membrane enriched in ganglioside GM1, cholesterol, and phosphatidylinositol 4,5 bisphosphate [PI(4,5)P_2_]. Such lipid rafts correspond to membrane areas stabilized by the presence of cholesterol within a liquid-ordered phase in which lateral diffusion of proteins and lipids is limited, resulting in the clustering of specific components (20). In neuroendocrine and various other secretory cells, lipid raft formation is necessary for the spatial organization of the exocytotic machinery including SNARE proteins (21–23). As a consequence, it is tempting to imagine that this lipid confinement at the exocytotic sites would help to prevent granular lipids and proteins from diffusing after secretory granules fusion with the plasma membrane.

Of particular interest, the formation of membrane domains corresponding to exocytotic sites is regulated by annexin-A2 (21, 24), a calcium- and phospholipid-binding protein involved in both exo- and endocytosis (25). Annexin-A2 has been described on clathrin-coated vesicles in the adrenal gland (26). The protein displays two typical YXX∅ endocytic motifs allowing its interaction with the μ2-subunit of the AP-2 complex that triggers clathrin recruitment (27). Therefore, annexin-A2 constitutes a strong candidate to participate in the coupling of secretory granule exocytosis with the subsequent compensatory endocytosis.

### PI(4,5)P2: Orchestrating exo-endocytosis coupling

Phosphatidylinositol 4,5 bisphosphate [PI(4,5)P_2_] has been largely described as an important regulator in exocytosis (28, 29) but it is also known to recruit and regulate multiple components involved in clathrin-mediated endocytosis. Therefore, the PI(4,5)P_2_-enriched membrane microdomains where exocytosis occurs is likely to constitute preferential spots for endocytosis, a perfect way to couple these two processes. For example, interaction of the adaptor protein AP-2 with YXX∅ endocytic motifs is driven by its interaction with PI(4,5)P_2_ (30). Local PI(4,5)P_2_ concentration regulates the membrane binding and deformation capacity of proteins containing Bin/amphiphysin/Rvs (BAR) domains including endophilin, syndapin, and amphiphysin, three membrane-deforming scaffold proteins that have been implicated in endocytotic processes (31, 32). Interestingly, it has been proposed that BAR domains participate in the fission of budding vesicles by synergistically cooperating with dynamin, a GTPase also sensitive to PI(4,5)P_2_ (33). Moreover, in collaboration with the group of Dr. Cardenas, we have recently demonstrated that dynamin-2 controls both exocytosis, by regulating fusion pore expansion, and the subsequent endocytosis of secretory granules in chromaffin cells (34). Finally, PI(4,5)P_2_ also regulates the dynamics of actin filaments, which are believed to limit plasma membrane protein diffusion and/or to directly participate in endocytosis (35, 36). Accordingly, we have previously demonstrated that actin filaments are formed at a post-docking step of exocytosis (37) and disruption of actin filament organization inhibits compensatory endocytosis after full fusion. These data suggest that actin remodeling is also implicated in the process of internalization *per se* (12).

### Phospholipid scrambling: A signal to trigger compensatory endocytosis?

One key feature of cell membranes is the asymmetric distribution of phospholipids between the leaflets. In the plasma membrane, phosphatidylserine (PS) and phosphatidylethanolamine (PE) reside in the inner cytoplasmic leaflet while phosphatidylcholine and sphingomyelin are located in the outer leaflet (38). In non-apoptotic cells, several biological functions are accompanied by a disruption of this phospholipid asymmetry resulting in the externalization of PS in the outer leaflet of the plasma membrane (39). This phenomenon has been observed during regulated exocytosis in mast cells (40), nerve terminals (41), and the neuroendocrine PC12 and chromaffin cells (12, 42, 43). More recently, we have shown that PS externalization occurs in specific domains at the frontier between the fused granule membrane and the plasma membrane and is triggered by the calcium-sensitive phospholipid scramblase-1 (PLSCR1). Interestingly, in chromaffin cells cultured from PLSCR1 knock-out mice, surface exposure of PS is not involved in exocytosis, but is required for granule membrane compensatory endocytosis (44).

To date no current evidence is available to explain the mechanism by which PS externalization is linked to compensatory endocytosis. However, two scenarios are possible. Firstly, as loss of phospholipid asymmetry can modify the mechanical stability of membranes (45), this might facilitates local reorganization of lipids surrounding the granule membrane transiently inserted within the plasma membrane and preserve the integrity of the granule membrane. Secondly, as an anionic phospholipid, PS confers negative charges and directly binds various proteins involved in exocytosis like annexin-A2, rabphilin, DOC 2, or synaptotagmin (46). PLSCR1-induced local decrease in PS concentration in the inner leaflet of the plasma membrane could therefore represent a signal to switch from exocytosis to endocytosis, thereby permitting the release of exocytotic components and/or the recruitment of the endocytic machinery.

However, whether externalization of PS simply reflects the loss of PS asymmetry or reveals more profound lipid reorganization is basically unknown and requires further investigation.

## How to Recapture a Large Dense Core Vesicle?

**Figure 2:**
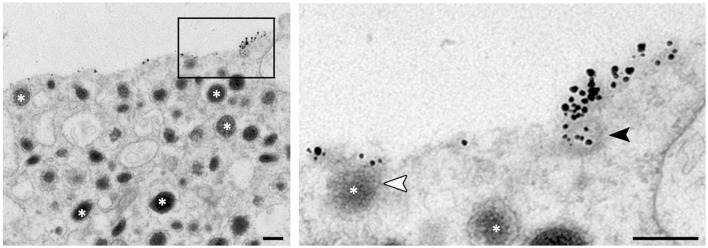
**Ultrastructural observation of budding vesicle from exocytotic spots in stimulated chromaffin cells**. To stain specifically the granule membrane fused with plasma membrane, stimulated cells are incubated in the presence of antibodies raised against the luminal region of dopamine-beta hydroxylase (DBH), a transmembrane marker of secretory granules (asterisks). Note that small vesicles budding can be observed from the DBH antibodies clusters (black arrowhead in enlarged view) suggesting a partial recapture of the granule membrane. The white arrowhead shows a granule fusing with the plasma membrane. Bar = 100 nm.

In chromaffin cells, we have found that clathrin is rapidly recruited to the granule membrane right after merging with the plasma membrane and that knocking-down clathrin expression drastically blocks compensatory endocytosis (12). The clathrin dependency of large dense core granule endocytosis raises the question of the mechanism by which a granule displaying a mean diameter of about 250 nm may be recaptured by a clathrin coat? Energetic constraints indicate that *in vitro* clathrin baskets assemble with a mean diameter of 100 nm or even less (80 nm) in the presence of adaptor proteins (47). In INS-1 insulinoma cells for example, the mean diameter of secretory granule ranges from 110 to 170 nm but the endocytotic events detected by capacitance measurements correspond to vesicles of 70 nm diameter (48). In mouse chromaffin cells, the average size of endocytic vesicles calculated from endocytic capacitance step sizes is 122 nm (49). Accordingly, recent electron microscopy experiments performed on chromaffin cells demonstrated that clathrin-coated vesicles size increases upon potassium stimulation (mean diameter of 87 nm) compared to resting cells (19). Altogether, these observations tend to demonstrate that large granule membranes may be recaptured as small pieces rather than a whole. Indeed, direct observations of the internalization of fused granule membranes allowed us to reveal early endocytotic events corresponding to 50–80 nm coated vesicles budding from granular membrane-bound proteins clusters (Figure 2).

## Endocytic Pathway

The intracellular route followed by the post-exocytotic internalized granules has not been fully characterized. The main difference with synaptic vesicle recycling is that, to be reused, large dense core granules need to be reloaded with matrix proteins, which most likely implies a re-maturation process involving the Golgi apparatus. The transient accessibility of granule-bound proteins at the cell surface during full fusion exocytosis has been exploited to label and follow post-fusion granules with specific antibodies or biotinylation. This approach was widely used in the 80s in order to demonstrate that granule-bound proteins transit through the Golgi region before being recycled in newly mature granules (10, 15, 50, 51). Alternatively, granule markers have been proposed to be degraded through a lysosomal pathway (52, 53). Both recycling and degradation pathways coexist and their proportion may depend on cell secretory activity. The recycling pathway leading to releasable granules is more predominant upon mild stimulation whereas above a certain threshold of membrane incorporation during intense exocytotic activity, the occurrence of bulk endocytosis will lead to the degradation of the internalized membrane (54, 55). Altogether, these data do not provide any information concerning the immediate fate of internalized granule membrane. Our group has observed that internalized granule-bound markers rapidly co-localize with the early endosomal marker EEA1, suggesting that chromaffin granule components might be retrieved through early endosomes after regulated exocytosis (12). Since clathrin is likely to retrieve the collapsed granule membrane as pieces and not as a whole, the early endosomes might constitute a transient sorting station that would sort the retrieved pieces to reconstitute a functional granule prior to entering the retrograde transport pathway to the TGN. Accordingly, we have observed budding of the immunogold-labeled DBH clusters present on endosomes (unpublished data).

## Conclusion

New evidence is now emerging to support the idea that, in neuroendocrine cells but also in neurons, vesicle/granule membranes do not intermix with the plasma membrane following full fusion exocytosis (56, 57). Compensation of membrane incorporation by endocytosis is a critical process and selective recapture of secretory organelles is required to maintain cellular homeostasis. Resolving the mechanisms that specifically preserve the granule membrane platform and retain granular components together after its incorporation in the plasma membrane to is the next challenging question to answer. Highly resolutive biophotonic approaches are now required to precisely investigate the dynamic behavior of secretory granules merged with the plasma membrane during and after exocytosis. Lipids clearly play a central role in this process, but attention should also be given to bi-functional proteins regulating both exo- and endocytosis, in particular annexin, synaptotagmin, and BAR domain-containing proteins.

## Conflict of Interest Statement

The authors declare that the research was conducted in the absence of any commercial or financial relationships that could be construed as a potential conflict of interest.
